# Trends and Patterns of Adverse Drug Reaction Reporting in Sierra Leone: A Retrospective Analysis of VigiFlow Data (2008–2022)

**DOI:** 10.1002/pds.70344

**Published:** 2026-03-10

**Authors:** Isatu Jalloh, Onome Thomas Abiri, Peter Bai James, Rajesh Vagiri, Neelaveni Padayachee

**Affiliations:** ^1^ Department of Pharmacy and Pharmacology, Faculty of Health Sciences University of the Witwatersrand Johannesburg South Africa; ^2^ Department of Clinical Pharmacy and Therapeutics, College of Medicine and Allied Health Sciences University of Sierra Leone Freetown Sierra Leone; ^3^ Department of Pharmacology and Therapeutics, College of Medicine and Allied Health Sciences University of Sierra Leone Freetown Sierra Leone; ^4^ Department of Pharmacovigilance and Clinical Trials, Pharmacy Board of Sierra Leone Freetown Sierra Leone; ^5^ National Centre for Naturopathic Medicine, Faculty of Health Southern Cross University, Pharmacy Board of Sierra Leone Lismore Australia; ^6^ Faculty of Pharmaceutical Sciences, College of Medicine and Allied Health Sciences University of Sierra Leone Freetown Sierra Leone; ^7^ Division of Pharmacology, Faculty of Pharmacy Rhodes University Makhanda South Africa

**Keywords:** adverse drug reactions, completeness score, individual case safety reports, pharmacovigilance, Sierra Leone, VigiFlow

## Abstract

**Purpose:**

Adverse drug reactions (ADRs) present significant obstacles for healthcare systems, impacting both patient safety and the effectiveness of treatments. Despite this, there is a scarcity of research on ADR reports in Sierra Leone, especially over long periods. This study aims to investigate the characteristics and reporting patterns found in the Sierra Leone pharmacovigilance database managed through VigiFlow.

**Method:**

This study analyzes reports of ADRs from Sierra Leone's national pharmacovigilance database, VigiFlow, spanning from January 2008 to December 2022. Data collected included patient demographics (age, sex), reporter characteristics (type of reporter, year of reporting), and ADR‐specific information (suspected medication, indication, ADR types (MedDRA), seriousness, outcome, actions taken, and time to onset), and completeness score. Descriptive statistics, chi‐square tests, and the Kruskal‐Wallis test with Bonferroni‐adjusted post hoc tests were applied to identify patterns and associations within the dataset.

**Results:**

A total of 3381 individual case safety reports (ICSRs) were analysed. The majority of reports involved females (54.7%) and adults aged 18 to 44 years (51.4%). Reporting rates increased after 2015, peaking in 2021. The most frequently implicated medications were anti‐infective drugs (40.7%) and antiparasitic medicines (34.1%), particularly ivermectin, albendazole, and vaccines for cholera and yellow fever. The most commonly reported ADRs were headache (13.2%), fever (12.2%), and diarrhoea (7.6%), primarily affecting the nervous system and general disorder classes. Pharmacists were responsible for 39.0% of reports and achieved the highest completeness score, with a mean of 0.78. Age was significantly associated with the seriousness, outcome, and onset time of ADRs (*p* < 0.001), while gender was significantly associated with onset time (*p* = 0.007).

**Conclusion:**

ADR reporting in Sierra Leone has improved, with antiparasitic medicines and vaccines most frequently linked to reactions. Sustaining progress requires enhanced training, public engagement, and strengthened active pharmacovigilance to ensure completeness and patient safety.

## Summary

- Most ADR reports involved adults aged 18–44 years and females, indicating higher reporting among women and young adults.
- Pharmacists and other healthcare professionals accounted for the majority of reports, reaffirming their central role in pharmacovigilance and the importance of strengthening reporting culture across all cadres.
- Anti‐infective and antiparasitic medicines, particularly ivermectin, albendazole, and vaccines (cholera, yellow fever), were most frequently suspected, reflecting the influence of large‐scale treatment and immunisation campaigns on national reporting trends.
- ADR reporting fluctuated substantially across the years, peaking in 2021 and reaching the lowest level in 2020, suggesting that public health emergencies such as COVID‐19 may disrupt routine pharmacovigilance activities.
- The completeness of reports was suboptimal, with missing data on dosage, indication, and seriousness criteria, underscoring the need for improved data quality assurance and feedback mechanisms.
- The most commonly reported ADRs were headache, pyrexia, diarrhoea, and pruritus, primarily non‐serious and self‐limiting, highlighting that most reported reactions were mild but remain critical for medicine safety monitoring.

## 1 Introduction

Adverse Drug Reactions (ADRs) are defined as harmful or unintended responses to medications that occur at doses typically used in humans for prophylaxis, diagnosis, or therapy [1]. ADRs range in severity from mild reactions to life‐threatening conditions [2, 3] and significantly affect patient outcomes by increasing morbidity, mortality, and healthcare costs [4, 5]. Globally, ADRs contribute substantially to healthcare expenditure and resource utilisation [6, 7]. Strengthening ADR monitoring and reporting is essential to improving patient safety, minimising preventable harm, and enhancing healthcare efficiency [8].

Pharmacovigilance, the science of detecting, assessing, understanding, and preventing adverse effects, plays a central role in ensuring the safe and rational use of medicines [9]. Weak pharmacovigilance fosters the spread of falsified medicines by delaying detection of ADRs and product‐quality defects, interrupting feedback to providers and consumers, and undermining trust in regulated supply chains. These factors are repeatedly highlighted in global analyses of pharmacovigilance gaps and counterfeit medicine risks [10, 11, 12, 13, 14]. Several sociodemographic, clinical, and systemic factors, such as age, sex, prescribing patterns, and genetic variability, can influence ADR occurrence and reporting [15, 16, 17].

In Africa, ADRs are most frequently reported among young adults due to higher medication exposure related to human immunodeficiency virus (HIV), tuberculosis, and pregnancy [18, 19, 20]. Severe ADRs can lead to prolonged hospitalisation, disability, or death, accounting for approximately 5% of hospital admissions and over 6% of inpatient cases [5, 21]. Despite this burden, ADR reporting remains low in many African countries [19], hindered by limited awareness, poor feedback mechanisms, and inadequate access to reporting tools [22, 23, 24, 25].

In Sierra Leone, the Pharmacy Board (PBSL), through the National Pharmacovigilance Centre (NPC), is mandated to ensure medicine safety in accordance with the Pharmacy and Drug Act of 2001 [26]. The PBSL initiated the establishment of the NPC in 2007 [27] and became the 87th member of the WHO's Program for International Drug Monitoring in 2008 [28]. That same year, Sierra Leone implemented VigiFlow, a web‐based national pharmacovigilance system linked to the WHO global database, VigiBase [28]. The system captures ADR reports through both passive (spontaneous) facility‐based reporting and active surveillance during mass drug administration campaigns and immunisation programmes [27, 28]. Reports submitted by healthcare professionals, field supervisors, or consumers are verified by trained pharmacovigilance officers and forwarded to VigiBase programmes [27, 28].

While pharmacovigilance activities have expanded across Africa [15, 28, 29, 30]. Few comprehensive analyses of Sierra Leone's ADR data exist. Existing studies have primarily focused on vaccines and antimicrobials [27, 31], highlighting inconsistent reporting trends and incomplete data. To address these gaps, this study conducted a retrospective analysis of ADR reports submitted to VigiFlow between 2008 and 2022. The objectives were to describe reporting trends and patterns, identify the most frequently suspected medicines, and assess report completeness to inform pharmacovigilance policy and capacity strengthening in Sierra Leone.

## 2 Method

### 2.1 Study Design

This quantitative longitudinal study employs a retrospective design to assess all ADR reports submitted to the Sierra Leone NPC documented in the VigiFlow. This approach helps identify temporal trends in ADRs, facilitates comparisons over time, and elucidates patterns that may inform clinical practice and policy.

### 2.2 Data Source

Data for the study were drawn from VigiFlow, which systematically records individual case safety reports (ICSRs) forwarded to the Sierra Leonean NPC. We focused on reports submitted between January 1, 2008, and December 31, 2022. The selection of this timeframe is critical, as it coincides with the establishment of the NPC in 2008, marking the beginning of structured ADR reporting in the country.

### 2.3 Data Extraction and Variable Specification

Data extraction was performed using specific parameters: patient demographics; details of suspected medications (including dosage and indication); onset timing of reactions; seriousness of ADRs; and actions taken. Detailed coding was conducted using the Medical Dictionary for Regulatory Activities (MedDRA) for consistent reporting and classification of adverse events, while medications were categorized using the WHO Drug Dictionary and the Anatomical Therapeutic Chemical (ATC) classification system.

### 2.4 Socio‐Demographic Variables

- Sex was recorded as either male or female on the ICSR form.
- Age was recorded in years and categorised as follows: Infants (0–23 months), Children (2–11 years), Adolescents (12–17 years), Adults (18–44 years), Middle‐aged (45–64 years), and Elderly (65 years and older).
- Year of report referred to the calendar year during which the ADR was submitted, from January 1 to December 31.
- Reporter type was classified according to the professional category of the individual submitting the report: physician, pharmacist, nurse, other healthcare professional, or consumer. Reports categorised as ‘unknown’ were included in the analysis to maintain the completeness of the national pharmacovigilance dataset, despite the reduced interpretability associated with this classification.

### 2.5 ADR Variables

ADR outcomes were classified as recovered, recovering, recovered with sequelae, not recovered, deceased, or unknown at the time of reporting, as determined by the reporter using the WHO‐predefined criteria. Time to onset was defined as the number of days between the initiation of the suspected treatment and the earliest recorded date of ADR onset. Suspected medications are defined as those identified by the reporter as possibly linked to an adverse reaction, without implying confirmed causality [8]. Medications were classified using the WHO Drug Dictionary and the Anatomical Therapeutic Chemical (ATC) Classification system [32]. ADRs and their indications were categorised according to the System Organ Class (SOC) hierarchy of MedDRA and coded as the Preferred Term (PT) [33]. The seriousness of ADRs was determined using WHO definitions, including death, life‐threatening events, hospitalisation or prolongation of hospitalisation, disability or incapacity, congenital anomaly, or other medically important conditions.

Completeness was evaluated using the VigiGrade criteria score automatically generated within VigiFlow, which operationalises the Bergvall et al. algorithm in accordance with WHO–UMC standards. VigiFlow applies domain‐specific penalties for missing or imprecise information across key pharmacovigilance variables, including patient demographics, suspected medication, reaction description, onset time, outcome, report type, dosage, country, primary reporter type, and comments. Each report receives a completeness score ranging from 0 to 1, where 0 indicates key variables missing or severely incomplete and 1 indicates a fully complete report. Higher values indicate greater data completeness [34]. The mean, median, and range of scores were calculated from the exported VigiGrade column in Excel and verified using the Excel Data Analysis ToolPak. Causality assessment was not conducted, as the study utilized secondary data from the national pharmacovigilance database (VigiFlow), which contains suspected ADRs reported by healthcare professionals and consumers.

### 2.6 Data Analysis

Statistical analyses were executed using Statistical Package for the Social Sciences (SPSS) version 30.0. The data were checked for missing values using descriptive statistics and frequency assessment in SPSS. All the missing data identified were coded accordingly. Variables were analysed according to the classification criteria described in the *Variable Specification* subsection (e.g., age groupings, reporter types, ATC medicine categories, MedDRA SOC/PT, WHO seriousness and outcome categories, and VigiGrade completeness scoring). Descriptive statistics, such as frequency counts and percentages, were used to summarise reporting trends across these categories. Normality tests pertaining to the data were conducted using a one‐sample Kolmogorov–Smirnov (KS) test. The association between socio‐demographic characteristics and ADR variables were assessed using the chi‐square test. Post hoc analyses were conducted using adjusted standardised residuals (ASRs) to identify categories contributing most to significant chi‐square associations, with absolute residual values greater than 1.96 considered significant (*p* < 0.05). The Kruskal‐Wallis H test was used to compare mean completeness scores (dependent variables) across reporters (independent variables), including physicians, pharmacists, other healthcare professionals, consumers/non‐healthcare professionals, and unknown reporters as completeness score did not meet normality assumptions. Pairwise post hoc comparisons were performed using Dunn's test. Dunn's test z‐statistics were used to assess differences between all possible reporter type pairs. Bonferroni‐adjusted post hoc comparisons were applied to examine pairwise differences where applicable. Any *p* < 0.05 was considered statistically significant.

## 3 Results

### 3.1 Sociodemographic and ADR Variables

During the study period, a total of 3381 reports were identified from the national pharmacovigilance database at the PBSL. Among these reports, patient sex was documented in 99.1% of cases, with 1848 (54.7%) females and 1502 (44.4%) males. Age data were available for 96.3% of the reports, with the 18–44‐year age group accounting for 1738 (51.4%) of the cases. In contrast, patients aged 65 years and older were the least affected, with only 91 reports (2.7%).

### 3.2 Type of Reporter

The type of reporter was noted in 3245 (96.0%) of the total reports (*n* = 3381). Among these, other healthcare professionals submitted the highest number of reports at 1604 (47.4%), while reports from consumers or non‐healthcare professionals accounted for the smallest segment, with only 80 (2.4%).

### 3.3 Onset Time of Reactions and Number of Reactions Per Report

The analysis of reaction onset times revealed that 1954 ADRs (57.8%) occurred within 24 h of drug administration (0–1 day). In contrast, 153 ADRs (4.5%) manifested between 2 and 7 days post‐administration, 62 ADRs (1.8%) were recorded between 8 and 30 days, and only 13 ADRs (0.4%) were reported after more than 30 days. Table 1 presents the sociodemographic and ADR variables of the sample. Of the 3381 ICSRs, 1498 (44.3%) documented one ADR, 967 (28.6%) noted two ADRs, 370 (10.9%) reported three ADRs, 360 (10.6%) indicated four ADRs, and 179 (5.3%) had five or more ADRs.

**TABLE 1:** Socio‐demographic characteristics of patients and reporting characteristics of ADR reports submitted in Sierra Leone (2008–2022).

Category	Level	*n* (%)	*p* *
Gender	Male	1502 (44.4)	< 0.001
	Female	1848 (54.77)	
	Unknown	31 (0.9)	
Age	28 days–23 months	125 (3.7)	< 0.001
	2–11 years	629 (18.6)	
	12–17 years	275 (8.1)	
	18–44 years	1738 (51.4)	
	45–64 years	398 (11.8)	
	≥ 65 years	91 (2.7)	
	Unknown	125 (3.7)	
Type of reporter	Physician	244 (7.2)	< 0.001
	Pharmacist	1317 (39.0)	
	Other healthcare professionals	1604 (47.4)	
	Consumer/non‐healthcare professional	80 (2.4)	
	Unknown	136 (4.0)	< 0.001
Onset time of reaction (days)	0–1	1954 (57.8)	
	2–7	153 (4.5)	
	8–30	62 (1.8)	
	> 30	13 (0.4)	
	Unknown	1199 (35.5)	
Number of reactions per report	1	1498 (44.3)	< 0.001
	2	967 (28.6)	
	3	370 (10.9)	
	4	360 (10.6)	
	≥ 5	179 (5.3)	
	Unknown	7 (0.2)	
Serious	No	3087 (91.3)	< 0.001
	Yes	183 (5.4)	
	Unknown	111 (3.3)	
Action taken	Dose not changed	593 (17.5)	< 0.001
	Dose reduced	19 (0.6)	
	Dose increased	2 (0.1)	
	Drug withdrawn	168 (5.0)	
	Not Applicable	1165 (34.5)	
	Unknown	1434 (42.4)	
Dose indicated	Indicated	2062 (61.0)	< 0.001
	Unknown	1319 (39.0)	
Start date indicated	Indicated	2265 (67.0)	< 0.001
	Unknown	1116 (33.0)	
Indications	Malaria	375 (11.1)	< 0.001
	Human immunodeficiency virus/acquired immunodeficiency syndrome	30 (0.9)	
	Cough and cold	6 (0.2)	
	Ascaris/Filariasis	609 (18.0)	
	Urinary tract infection/sexually transmitted infection	8 (0.2)	
	Cholera Immunisation	212 (6.3)	
	Coronavirus disease	8 (0.2)	
	Psychosis	4 (0.1)	
	Hypertension	4 (0.1)	
	Fever	5 (0.1)	
	Induced Labour	4 (0.1)	
	Respiratory Infection	4 (0.1)	
	Measle immunisation	98 (2.9)	
	Tuberculosis	22 (0.7)	
	Schizophrenia	6 (0.2)	
	Vomiting	5 (0.1)	
	Yellow fever	136 (4.0)	
	Schistosomiasis	14 (0.4)	
	Others	36 (1.1)	
	Unknown	1795 (53.1)	

* One‐sample Kolmogorov–Smirnov (K–S) test. Others include indications reported fewer than five times each (e.g., abdominal pain, allergy, anemia, diarrhea, Ebola immunisation, headache, hypotension, poliomyelitis, pre‐eclampsia, typhoid).

### 3.4 Dose and Action Taken

The dose was reported for 61% of cases (*n* = 3381). Actions taken regarding the reported ADRs were documented in 782 (23.1%) of the cases. Notably, the dose remained unchanged in 593 (17.5%) of these events where action was specified. The suspect drug was withdrawn in 168 (5.0%) cases, reduced in 19 (0.6%), and increased in 2 (0.1%) cases. For the remaining 2599 (76.9%) ADRs, the actions taken were recorded as “unknown” or “not applicable”. “Not applicable” indicates cases where no action was relevant, such as when treatment had already been completed, continuation was unnecessary, or medicines were administered as part of mass drug administration campaigns or immunisation programmes, where withdrawal or dose adjustment would not be relevant. “Unknown” refers to reports where the action field was left blank or unspecified.

### 3.5 Indications for Use

Of the 3381 entries for suspected medicines, 1586 (46.9%) included information on indications for use. A total of 35 distinct indications were reported, with Ascaris/filariasis being the most prevalent at 609 (18.0%), followed by malaria at 375 (11.1%), cholera immunisation at 212 (6.3%), and yellow fever at 136 (4.0%). The “Others” category for indications encompasses conditions with fewer than five reports each, collectively accounting for only a minimal percentage of the dataset. This category included a diverse set of conditions such as abdominal pain, accidental exposure to a substance, allergy, anaemia, anaesthetic, blood transfusion, congestive heart failure, contraception, diarrhoea, drug poisoning, Ebola immunisation/prophylaxis, energy drink abuse, headache, hypotension, pain, peptic ulcer, poliomyelitis, pre‐eclampsia, and typhoid.

### 3.6 Suspected Medicines

A total of 4408 suspected medicines were identified throughout the study period, encompassing all 14 ATC groups. The frequencies of the top 20 suspected medicines are as follows: Ivermectin was the most frequently reported medicine, comprising 841 (19.1%) of all ICSRs, followed by Albendazole at 721 (16.4%), and the Cholera vaccine at 648 (14.7%). Notably, some ICSRs contained multiple suspected medicines, leading to classification as separate entries for analytical purposes. (Table 2).

**TABLE 2:** Suspected Medicines, ADRs by MedDRA Preferred Terms, Seriousness of ADRs, and Outcomes.

Category	Level	*n* (%)
Top suspected medicines (*n* = 4408 suspected medicines)	Ivermectin	841 (19.1)
	Albendazole	721 (16.4)
	Cholera vaccine	648 (14.7)
	Yellow fever vaccine	594 (13.5)
	Amodiaquine + Artesunate	353 (8.0)
	Albendazole + Ivermectin	211 (4.8)
	Measles + Rubella vaccines	145 (3.3)
	COVID‐19 vaccine	124 (2.8)
	Measles vaccine	98 (2.2)
	Paracetamol	48 (1.1)
	Praziquantel	42 (1.0)
	Polio vaccine	37 (0.8)
	Lamivudine, Nevirapine, Zidovudine	23 (0.5)
	Sulfamethoxazole, Trimethoprim	22 (0.5)
	Amoxicillin	18 (0.4)
	Artesunate	15 (0.3)
	Quinine	14 (0.3)
	Amodiaquine	14 (0.3)
	Diclofenac	13 (0.3)
	Lamivudine, Nevirapine, Stavudine	12 (0.3)
Top 20 MedDRA Preferred Terms (*n* = 6053)	Headache	797 (13.2)

Pyrexia	741 (12.2)

Diarrhoea	462 (7.6)

Dizziness	443 (7.3)

Pruritus	407 (6.7)

Vomiting	363 (6.0)

Myalgia	223 (3.7)

Abdominal pain	220 (3.6)

Rash	189 (3.1)

Asthenia	184 (3.0)

Nausea	178 (3.0)

Musculoskeletal weakness	171 (2.8)

Urticaria	138 (2.3)

Somnolence	98 (1.6)

Oedema	78 (1.3)

Pain	74 (1.2)

Abdominal pain upper	60 (1.0)

Decreased appetite	60 (1.0)

Arthralgia	58 (1.0)

Cough	54 (0.9)
Seriousness criteria (*n* = 3381 reports)	Other medically important conditions	16 (0.5)
	Disabling/Incapacitating	8 (0.2)
	Caused/prolonged hospitalisation	64 (1.9)
	Life threatening	84 (2.5)
	Death	9 (0.3)
	Unknown	3200 (94.6)
Outcome of ADR (3381)	Recovered	2391 (70.7)
	Recovered with sequelae	6 (0.2)
	Recovering	786 (23.2)
	Not recovered	16 (0.5)
	Died	11 (0.3)
	Unknown	171 (5.1)

Abbreviations: MedDRA = Medical Dictionary for Regulatory Activities, ADR = Adverse Drug Reaction, Medicines separated by a plus sign (+) represent fixed‐dose combinations or medicines administered together as part of a combined regimen. Medicines separated by commas represent individual medicines reported concurrently but not formulated as fixed‐dose combinations.

### 3.7 ADRs By MedDRA Preferred Terms

In terms of MedDRA preferred terms, headache emerged as the most frequently reported term, accounting for 797 cases (13.2%) of all ICSRs during the study period. Following this, pyrexia was reported in 741 cases (12.2%), diarrhoea in 462 cases (7.6%), and dizziness in 443 cases (7.3%). (Table 2).

### 3.8 Seriousness of ADRs

Among the 3381 reports received, 5.4% were classified as serious. Of the total reports, 181 (5.4%) contained information on the seriousness criteria. Life‐threatening reactions were the most frequently cited reason for seriousness classification, with 84 (2.5%), followed by prolonged hospitalisation in 64 (1.9%). Other medically important conditions were recorded in 16 (0.5%), and instances classified as disabling/incapacitating 8 (0.2%) and those resulting in death numbered 9 (0.3%). (Table 2).

### 3.9 Outcomes of ADRs

Data regarding the outcomes of ADRs were available for 3381 ICSRs. A significant majority of patients, 2391 (70.7%), achieved full recovery, while 786 (23.2%) were reported as still recovering at the time of reporting. Additionally, 16 (0.5%) had not yet recovered from their ADRs, and fatal outcomes were reported in 11 (0.3%). (Table 2).

### 3.10 Suspected Medicines Per ATC Group

The suspected medicines reported were distributed across all 14 ATC groups, with a cumulative total of 4408 medicines identified during the review. The most reported ATC group was Anti‐infectives for systemic use (1795 cases, 40.7%), followed by Anti‐parasitic products, insecticides, and repellents (1503 cases, 34.1%), dermatological preparations (881 cases, 20.0%), and Alimentary tract and metabolism (49 cases, 1.1%). (Table 3).

**TABLE 3:** Suspected medicines per ATC classification, and ADRs according to SOC class.

Characteristics	Level	*n* (%)
ATC Level 1 Class of Suspected Medicines (*n* = 4408)	Anti‐infectives for systemic use	1795 (40.7)
	Anti‐parasitic products, insecticides and repellents	1503 (34.1)
	Dermatologicals	881 (20.0)
	Alimentary tract and metabolism	49 (1.1)
	Nervous system	38 (0.9)
	Sensory organs	37 (0.8)
	Respiratory system	29 (0.7)
	Genitourinary system and sex hormones	20 (0.5)
	Musculoskeletal system	18 (0.4)
	Cardiovascular system	15 (0.3)
	Blood and blood‐forming organs	9 (0.2)
	Various	7 (0.2)
	Antineoplastic and immunomodulating agents	6 (0.2)
	Systemic hormonal preparations	1 (0.0)
System Organ Class (SOC) of Reported ADRs (*n* = 5242)	Nervous system disorders	1241 (23.7)

General disorders and administration site conditions	1216 (23.2)

Gastrointestinal disorders	1092 (20.8)

Skin and subcutaneous tissue disorders	717 (13.7)

Musculoskeletal and connective tissue disorders	464 (8.9)

Respiratory, thoracic and mediastinal disorders	83 (1.6)

Metabolism and nutrition disorders	76 (1.4)

Psychiatric disorders	67 (1.3)

Infection and infestation	51 (1.0)

Eye disorders	43 (0.8)

Ear and labyrinth disorders	39 (0.7)

Immune system disorders	38 (0.7)

Cardiac disorders	28 (0.5)

Renal and urinary disorders	23 (0.4)

Reproductive system and breast disorders	17 (0.3)

Blood and lymphatic system disorders	15 (0.3)

Vascular disorders	15 (0.3)

Investigations	7 (0.1)

Hepatobiliary disorders	7 (0.1)

Injury, poisoning and procedural disorders	3 (0.1)

*Note:* “Various” corresponds to ATC Level 1 code V, which includes medicines that do not fall under other specific anatomical groups (e.g., diagnostic agents and miscellaneous products). “Investigations” is a valid MedDRA System Organ Class (SOC) capturing laboratory and diagnostic abnormalities rather than clinical symptoms.

Abbreviations: ATC = Anatomical Therapeutic Chemical classification; SOC = System Organ Class.

### 3.11 ADRs By System Organ Classification

The 3381 ICSRs accounted for 5242 total ADRs. This study identified the top 20 leading ADRs categorised by SOC. Among the reported reactions, nervous system disorders emerged as the most frequently reported SOC, with 1241 (23.7%), followed closely by General disorders and administration site conditions at 1216 (23.2%). Gastrointestinal disorders accounted for 1092 (20.8%), and skin and subcutaneous tissue disorders at 717 (13.7%). (Table 3).

### 3.12 Trends in Adverse Drug Reactions

**FIGURE 1:**
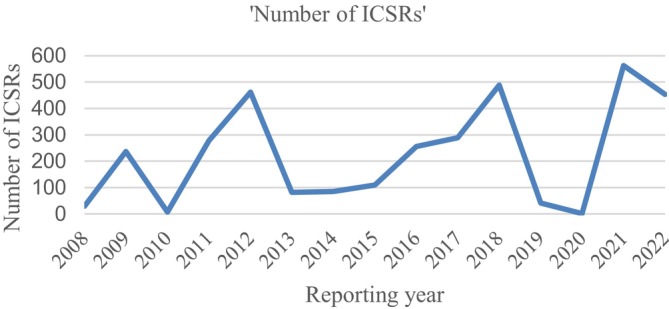
Yearly distribution of ADR reports.

The analysis of ADR reporting trends revealed that 2021 had the highest number of reported ADRs, accounting for 16.7% of the total reports. This was followed by 2018 with 14.5%, 2012 with 13.7%, and 2022 with 13.4%. Notably, there was a considerable increase in reporting from 2015 to 2018, whereas the lowest rate of reporting occurred in 2020 (Figure 1).

### 3.13 Completeness of ADR Reporting

The completeness score for the reports received ranged from 0.15 to 1, with a mean of 0.65; only 28.16% of the reports achieved a completeness score greater than 0.8. In this study, patient age and sex were missing from 3.7% and 0.9% of the reports, respectively. Furthermore, indications, doses, and reporter types were absent in 53.1%, 39.0%, and 4.0% of cases, respectively. Information regarding the action taken with the suspected drug was unknown or not applicable in 76.9% of the cases, and the outcome was undisclosed in 5.1% of instances. Additionally, only 64.5% of time‐to‐onset calculations could be performed, as 35.3% of the cases lacked this critical information.

### 3.14 Association Between Socio‐Demographics and ADR Variables

Table 4 shows that socio‐demographic characteristics, particularly age and reporter type, are significantly associated with ADR seriousness, action taken, outcome, onset time, and number of ADRs per report, whereas gender is only associated with onset time. To identify which categories drive these multi‐group associations, Bonferroni‐adjusted post hoc analyses were performed using adjusted standardised residuals (Table S1–S3).

**TABLE 4:** Association between age, gender, reporter type and ADR variables.

ADR Variable	Category	Frequency (%)	Age (p‐value)	Gender (p‐value)	Reporter Type (p‐value)
Seriousness criteria	Serious	183 (5.4)	< 0.001*	0.812	< 0.001*
	Non‐serious	3087 (91.3)			
	Unknown	111 (3.3)			
Action taken	Drug withdrawn	168 (5.0)	< 0.001*	0.454	< 0.001*
	Dose not changed	593 (17.5)			
	Dose modified (Dose increased + Dose reduced)	21 (0.6)			
	Not applicable	1165 (34.5)			
	Unknown	1434 (42.4)			
Outcome of ADR	Recovered	2391 (70.7)	0.021*	0.653	< 0.001*
	Recovering	786 (23.2)			
	Recovered with sequelae	6 (0.2)			
	Not recovered	16 (0.5)			
	Died	11 (0.3)			
	Unknown	171 (5.1)			
Onset time of reaction (days)	0–1	1954 (57.8)	< 0.001*	0.007*	< 0.001*
	2–7	153 (4.5)			
	8–30	62 (1.8)			
	> 30	13 (0.4)			
	Unknown	1199 (35.5)			
Number of ADRs per report	1	1498 (44.3)	< 0.001*	0.058	< 0.001*
	2	967 (28.6)			
	3	370 (10.9)			
	4	360 (10.6)			
	≥ 5	179 (5.3)			
	Unknown	7 (0.2)			

* Chi‐square test of association used for all categorical variables. Post hoc comparisons were examined using adjusted standardised residuals with Bonferroni‐adjusted significance thresholds; *p* ≤ 0.05.

After Bonferroni correction, infants (28 days–23 months) and elderly patients (≥ 65 years) remained over‐represented in life‐threatening ADRs and deaths, while adults 18–44 years were under‐represented in these severe categories and more often fully recovered, indicating age vulnerability. Physicians disproportionately reported hospitalisations and fatalities; other healthcare professionals reported more life‐threatening events, and pharmacists showed no over‐ or under‐representation in specific seriousness categories despite high reporting volume. Consumers were more likely to report ongoing reactions, and pharmacists most often submitted single‐ADR reports, whereas other healthcare professionals, physicians, and consumers more frequently documented multiple ADRs per report.

For onset time, Bonferroni‐adjusted residuals showed that females and adults 18–44 years were over‐represented among immediate reactions (0–1 day), while males and younger children more often had delayed or unknown onset. Overall, the Bonferroni‐corrected post hoc results confirm that the significant chi‐square tests in Table 4 arise from specific age and reporter subgroups rather than uniform differences across all categories.

The Kruskal‐Wallis test revealed a highly significant difference in completeness scores among the various reporter types (H = 569.68; *p* < 0.001). This finding indicates that reporting completeness varies substantially by reporter type. Post hoc Dunn's test identified statistically significant differences (*p* < 0.05) between most pairs of reporter types except for pharmacists and other healthcare professionals (*z* = −0.80). (Table 5).

**TABLE 5:** Association between reporter type and completeness score.

ADR variable		Completeness mean score (±SD)	*p* *
Reporter type	Physicians	0.54 (±0.29)	< 0.001
	Pharmacists	0.78 (±0.22)	
	Other healthcare professionals	0.58 (±0.28)	
	Consumers/non‐ healthcare professionals	0.60 (±0.12)	
	Unknown	0.44 (±0.25)	
	Total	0.65 (±0.27)	

* Kruskal‐Wallis H test used to compare completeness scores across reporter types. The “Total” row is presented for descriptive purposes only and was not included in the statistical test.

## 4 Discussion

### 4.1 Summary of Main Findings

The analysis of 15 years of pharmacovigilance data from Sierra Leone's VigiBase revealed essential trends and disparities in ADR reporting. The number of reports fluctuated significantly from year to year, where it peaked in 2021 and was at the lowest point in 2020. The medicines most often linked to ADRs were anti‐infectives, such as ivermectin and albendazole, as well as vaccines used in mass drug administration campaigns. More than 70% of the patients recovered. Still, the reports were often missing important details. Few reports came directly from patients. Pharmacists tended to submit more complete reports than other types of reporters.

Most ADRs involved women and adults aged 18 to 44. A pronounced predominance of ADR reports among female patients compared with male patients aligns with findings from various studies conducted in different regions [16, 27, 35, 36]. Despite this trend, some studies, including those in Nigeria and South Africa, suggest that men may experience higher overall rates of some ADRs [15, 37, 38] or that there may be no significant difference in reporting across sexes [20, 39]. This higher reporting rate among females is sometimes attributed to physiological differences that influence drug metabolism and response [40, 41]. Factors such as hormonal influences, body composition variances, and differing pharmacokinetics may contribute to an increased likelihood of females experiencing ADRs, indicating the need for tailored pharmacotherapy approaches [42, 43]. Additionally, women tend to demonstrate higher health‐seeking behavior [44].

Our results also indicate that reporting patterns among age groups align with global trends, particularly showing higher ADR prevalence in the economically active population aged 18–44 years [15, 18, 19]. The strong associations between age groups and variables such as seriousness criteria, actions taken, and onset time of reactions highlight the age‐specific vulnerabilities, particularly among younger and older populations. This predominance may reflect higher levels of healthcare utilisation and medicine exposure among adults aged 18–44 years, particularly during public health campaigns and routine service delivery in Sierra Leone [45].

The analysis of reporting sources reveals that healthcare professionals generated most of the reports, with nurses and community health officers leading, followed closely by pharmacists. In contrast, reports from consumers were minimal, reflecting similar findings in Nigeria [20]. Consumer reporting was substantially lower compared to data from Denmark, the Netherlands, and Sweden [46] indicating limited public engagement in pharmacovigilance. In our study, the high percentage of reports from healthcare professionals, particularly other healthcare professionals, exceeded those reported in South Africa (26.50%), Nigeria (12%), and Australia (5.7%) [20, 36, 47]. The strong link between reporter type and other variables, such as report completeness, suggests that pharmacists, who demonstrate superior reporting quality, should be more actively involved in pharmacovigilance training and supervision. Pharmacists, as the most accessible healthcare professionals, play a pivotal role in the detection and reporting of ADRs, promoting drug safety [48]. Despite these insights, the overall low completeness score indicates systemic gaps in reporting. Targeted education, digital reporting tools, and public sensitization are vital to enhancing engagement in this domain.

Moreover, the mean completeness score for ADR reports was only 0.65, with just 28.16% achieving a score above 0.8, indicating significant insufficiencies in documentation. Although this is an improvement over previous findings in South Africa, where only 11.29% of reports were complete [36], the rate remains below the 44% completeness observed in a Sierra Leone study analysing antimicrobial reports from 2017 to 2021 [27]. The absence of critical data jeopardises comprehensive assessments necessary for effective signal detection, underscoring the need to improve ADR reporting completeness to bolster pharmacovigilance efforts and enhance patient safety. *The pattern of medications and ADRs observed underscores the need for targeted risk‐management strategies. Anti‐infectives for systemic use, particularly Ivermectin, Albendazole, and cholera vaccine, were most frequently implicated, with nervous system, administration site, gastrointestinal, and skin disorders predominating*. Headache was the leading preferred term and was most commonly associated with Ivermectin, Albendazole, and vaccines used in mass drug administration programmes. *Most reports involving these medicines resulted in recovery. Deaths were rare (0.3%) and were reported mainly in association with anti‐infective antiparasitic medicines, with over 70% of patients achieving full recovery. The low proportion of reports with seriousness classification (5.4%) limits the timely detection of severe outcomes*. Strengthening documentation, increasing pharmacist‐led training, and integrating pharmacovigilance into mass drug‐administration campaigns should be a priority to enhance report completeness, facilitate faster signal detection, and safeguard public health. In terms of medications associated with ADRs, the most frequently reported were Ivermectin, followed by Albendazole and the cholera vaccine. In contrast studies from other settings have shown different leading suspected drugs, in Ghana Amodiaquine was the most frequently reported followed by Pyrimethamine and Sulfadoxine [16]. In South Africa, the most reported suspected drugs were Interferon Beta‐1a, Enalapril, and Darbepoetin Alfa [36], and in Ethiopia Trimethoprim ‐Sulfamethoxazole, Amoxicillin, and Zidovudine predominated [49]. These discrepancies in drug reporting may stem from variations in prescribing practices, healthcare system structures, and higher reporting in public health campaigns that have incorporated pharmacovigilance as an integral part of their operations. Notably, the predominant group of reported medications consists of anti‐infectives for systemic use, followed by anti‐parasitic products, insecticides, repellents, dermatological agents, and drugs for the alimentary tract and metabolism. Similar patterns have been observed in Colombia, Portugal, Saudi Arabia, and other African nations, where similar classes were also identified as frequently associated with ADRs [19, 36, 50, 51, 52]. The prominence of anti‐infectives, especially those associated with mass drug administration campaigns in Sierra Leone, highlights how health interventions can influence reporting practices and underscores the importance of integrating pharmacovigilance into public health strategies [27, 28]. Within the top ten reported suspected medicines, most were used for the management of helminthic and parasitic infections, with Ascaris and filariasis identified as the most common indications. This trend likely reflects the mass drug administration campaigns in Sierra Leone aimed at deworming and malaria prevention, which are coupled with active reporting policies associated with such campaigns [27, 28]. Additionally, the increased reporting of these medications can be attributed to their availability without a prescription and the frequent assumption that experienced symptoms are malaria‐related, prompting immediate treatment.

Categories of ADRs reported primarily included nervous system disorders, general disorders and administrative site conditions, gastrointestinal disorders, and skin and subcutaneous tissue disorders, findings consistent with earlier research from Ghana and South Africa [15, 36]. A global analysis likewise identified that general and administration site conditions, skin and subcutaneous tissue disorders, and nervous system disorders were among the most frequently reported [35]. Headaches emerged as the most preferred term in our dataset, consistent with post‐treatment or post‐vaccination reactions with previous reports identifying headache, pyrexia, diarrhoea, and dizziness as common side effects [15, 20]. Interestingly, a comparison with data from Japan showed that headaches were reported less often in many other countries [53], a difference that may reflect cultural perceptions of symptoms or variations in how reports are collected. In our study, only 5.4% of the ICSRs included information regarding seriousness criteria, highlighting a concerning underreporting trend reflected in studies from Nigeria [20, 54, 55], and sharply contrasting with South African research that reported a seriousness classification rate of 55.9% [36]. Given the significant implications of severe ADRs for patient health, the national pharmacovigilance unit must promote robust reporting practices and meticulously analyse these reports for effective drug safety monitoring.

Among the 3381 reported ICSRs, 94.9% contained information on outcomes, with only 0.3% resulting in death. The reporting of some fatal cases highlights the need for better monitoring. Most patients reported full recovery, mirroring higher recovery rates reported in Ghana and Sierra Leone [15, 56]. In contrast, a retrospective review in Nigeria indicated that only 42% of patients reported either full recovery or were in the process of recovering from serious ADRs when reported [20].

Overall, trends in ADR reporting from 2008 to 2022 show inconsistency, with the fewest reports submitted in 2020, likely reflecting the impact of the COVID‐19 pandemic on healthcare services and reporting capabilities during that year. The significant increase in reports from 2015 to 2018, coinciding with mass deworming campaigns utilising Ivermectin and Albendazole, indicates how public health initiatives can enhance ADR reporting and awareness. Similar patterns have been documented in other African nations, where pharmacovigilance efforts have been effectively integrated into public health campaigns [57, 58, 59].

### 4.2 Implications for Policy and Practice

The study's findings convey important implications for pharmacovigilance policy and practice in Sierra Leone. The predominance of female patients and young adults among ADR reports suggests a need for targeted educational strategies that address demographic‐specific risk factors. Policymakers should prioritize initiatives that emphasise these population characteristics to boost awareness and reporting rates. With a high volume of reports generated by healthcare professionals, comprehensive training programs are crucial for enhancing their ADR reporting knowledge. Given the low consumer engagement in reporting, public education initiatives, and community outreach, an accessible online reporting portal is essential to fostering a culture of safety. Furthermore, enhancing the completeness of ADR reports is critical for effective signal detection and ensuring patient safety. Strategies to achieve this include mandating completion of key data fields, implementing automated prompts, and conducting regular staff training. Regulatory focus on data quality and regular audits can incentivise thorough documentation and strengthen the pharmacovigilance framework.

### 4.3 Recommendations for Further Research

Further research should focus on identifying barriers to ADR reporting, especially among consumers, through qualitative studies that explore perceptions and awareness. Longitudinal studies are needed to assess the impact of educational interventions or policy changes on reporting patterns. Continuous monitoring of ADR trends is vital, particularly for serious cases, to understand the causal relationships between medications and adverse effects. Collaboration with regional and international pharmacovigilance organisations can enhance data comparability and promote shared learning.

## 5 Limitations

This study has several limitations. Because of the retrospective nature, we cannot draw conclusions about cause and effect. Many reports lacked key information, including severity, action taken, dose, indication, and reaction onset time. We often lacked data on why the drug was given. When this was available, it mostly related to infectious diseases and vaccination campaigns. This suggests most data come from mass drug administration programs. As a result, applying these findings to regular healthcare settings is difficult. We used only basic statistical tests, such as the chi‐square test, and did not adjust for other potential influencing factors. Therefore, our associations are descriptive, not causal. There may be underreporting from consumers, more reports from healthcare professionals, and a lack of follow‐up data. This makes it hard to assess long‐term outcomes. These issues limit the extent to which we can generalise the results to other African or low‐ and middle‐income countries. Still, our findings provide a broad overview of how adverse drug reactions are reported and the trends in Sierra Leone.

## 6 Conclusion

The findings reveal significant trends in ADR reporting, including a higher prevalence among female patients and younger adults aged 18–44. The predominance of reports related to anti‐infectives, particularly medications used for helminthic and parasitic infections, underscores the influence of mass drug campaigns and the increased accessibility of these medications.

Despite the thorough nature of the data, the study identified notable limitations, including incomplete reporting and low consumer engagement in ADR reporting. These results emphasize the critical need for enhanced pharmacovigilance measures to ensure patient safety and improve the quality of healthcare delivery in Sierra Leone.

## 7 Plain Language

This study examined reports of adverse drug reactions (ADRs) in Sierra Leone over a 15‐year period, using information from the national database called VigiFlow. ADRs are harmful effects caused by medications and vaccines, and monitoring them is crucial for patient safety. Our findings revealed that women and young adults were the most likely to report ADRs, particularly related to anti‐infective drugs like Ivermectin and Albendazole, which are often used in public health campaigns. Despite a substantial number of reports, we found that many lacked important details, indicating that drug safety monitoring could be significantly improved. We recommend better training for healthcare providers to enhance reporting practices, as well as initiatives to encourage patients to share their experiences with medications. By improving these reporting processes, we can better protect patient health and inform safer drug use in Sierra Leone and other similar settings.

## Funding

The authors have nothing to report.

## Ethics Statement

This study received ethical clearance from the University of Witwatersrand Human Research Ethics Committee (Medical) under Clearance Certificate No. M230804 M240312‐A‐0001, as well as from the Sierra Leone Ethics and Scientific Review Committee.

## Conflicts of Interest

The authors declare no conflicts of interest.

## Supporting information


**Data S1:** pds70344‐sup‐0001‐Tables.docx.
