# Supplementary material for: Trends and Patterns of Adverse Drug Reaction Reporting in Sierra Leone: A Retrospective Analysis of VigiFlow Data (2008–2022)
Source: Pharmacoepidemiol Drug Saf. 2026 Mar 10;35(3):e70344. doi: 10.1002/pds.70344 (PMC12975695; doi:10.1002/pds.70344)
Supplement: Supplementary file 1 — Data S1: pds70344‐sup‐0001‐Tables.docx. [file PDS-35-e70344-s001.docx]

**Supplementary Material**

**Supplementary Table S1: Adjusted Standardized Residuals (ASR) for Significant Associations Between Age Groups and ADR Variables**

**Association: Age Group × Seriousness Criteria (χ² p < 0.001)**

| Age Group | Life-threatening | Death | Hospitalization | Non-serious |
| --- | --- | --- | --- | --- |
| Infants (28 days–23 months) | **+3.2*** | **+2.8*** | +0.8 | -1.5 |
| Children (2–11 years) | +0.4 | -0.2 | -0.6 | +0.3 |
| Adolescents (12–17 years) | -0.9 | -0.5 | -0.3 | +0.5 |
| Adults (18–44 years) | -1.2 | -1.8 | -0.4 | +1.4 |
| Middle-aged (45–64 years) | +0.6 | +0.3 | +1.2 | -0.8 |
| Elderly (≥65 years) | **+4.1*** | **+3.5*** | +1.6 | **-2.3*** |

**Association: Age Group × Action Taken (χ² p < 0.001)**

| Age Group | Dose unchanged | Drug withdrawn | Not applicable |
| --- | --- | --- | --- |
| Infants (28 days–23 months) | +0.6 | -0.4 | -0.2 |
| Children (2–11 years) | **+2.4*** | -0.8 | -1.1 |
| Adolescents (12–17 years) | +0.8 | +0.2 | -0.5 |
| Adults (18–44 years) | **-2.8*** | +1.5 | +1.9 |
| Middle-aged (45–64 years) | +0.4 | +0.6 | +0.1 |
| Elderly (≥65 years) | -0.3 | -0.2 | +0.5 |

**Association: Age Group × Outcome (χ² p = 0.021)**

| Age Group | Recovered | Recovering | Not recovered | Died |
| --- | --- | --- | --- | --- |
| Infants (28 days–23 months) | -0.8 | +1.2 | +0.4 | +1.3 |
| Children (2–11 years) | -1.6 | **+2.6*** | +0.2 | -0.5 |
| Adolescents (12–17 years) | -0.4 | +0.9 | -0.3 | +0.1 |
| Adults (18–44 years) | **+2.1*** | -1.8 | -0.6 | -0.9 |
| Middle-aged (45–64 years) | +0.5 | -0.7 | +0.8 | +0.2 |
| Elderly (≥65 years) | -0.9 | -1.1 | +0.5 | +1.4 |

**Association: Age Group × Onset Time (χ² p < 0.001)**

| Age Group | 0–1 day | 2–7 days | 8–30 days | >30 days | Unknown |
| --- | --- | --- | --- | --- | --- |
| Infants (28 days–23 months) | -0.5 | +0.8 | +0.3 | -0.2 | +0.6 |
| Children (2–11 years) | -1.3 | +1.6 | **+2.2*** | +0.8 | +0.9 |
| Adolescents (12–17 years) | +0.6 | +0.4 | +0.5 | +0.2 | -0.8 |
| Adults (18–44 years) | **+3.4*** | -0.9 | -1.2 | -0.6 | **-2.1*** |
| Middle-aged (45–64 years) | +0.2 | -0.3 | -0.4 | +0.3 | +0.5 |
| Elderly (≥65 years) | -1.2 | -0.6 | +0.2 | +0.4 | +1.3 |

**Association: Age Group × Number of ADRs per Report (χ² p < 0.001)**

| Age Group | 1 ADR | 2 ADRs | 3 ADRs | 4 ADRs | ≥5 ADRs |
| --- | --- | --- | --- | --- | --- |
| Infants (28 days–23 months) | +0.8 | -0.5 | -0.3 | -0.6 | +0.2 |
| Children (2–11 years) | -1.5 | +0.8 | **+2.7*** | +1.2 | -0.4 |
| Adolescents (12–17 years) | **+2.3*** | -0.9 | -1.1 | -0.8 | -0.5 |
| Adults (18–44 years) | +0.9 | -0.6 | -1.4 | -0.3 | +0.8 |
| Middle-aged (45–64 years) | -0.7 | +1.2 | +0.5 | +0.8 | +0.4 |
| Elderly (≥65 years) | -0.4 | +0.3 | -0.6 | +0.9 | -0.2 |

**Supplementary Table S2: Adjusted Standardized Residuals (ASR) for Significant Associations Between Reporter Type and ADR Variables**

**Association: Reporter Type × Seriousness Criteria (χ² p < 0.001)**

| Reporter Type | Life-threatening | Death | Hospitalization | Other serious | Non-serious |
| --- | --- | --- | --- | --- | --- |
| Physician | +1.5 | **+3.2*** | **+2.8*** | +0.8 | -1.9 |
| Pharmacist | -0.8 | -0.6 | -0.4 | -0.5 | +0.6 |
| Other HCP | **+2.4*** | +0.9 | +1.3 | +1.1 | -1.2 |
| Consumer/non-HCP | +0.6 | +0.4 | -0.3 | +0.2 | -0.5 |
| Unknown | -1.2 | -0.8 | -1.5 | -0.6 | +1.3 |

**Association: Reporter Type × Outcome (χ² p < 0.001)**

| Reporter Type | Recovered | Recovering | Not recovered | Died |
| --- | --- | --- | --- | --- |
| Physician | **+2.6*** | -1.8 | +0.5 | **+2.1*** |
| Pharmacist | **+2.3*** | -1.5 | -0.4 | -0.7 |
| Other HCP | -1.4 | +1.2 | +0.2 | +0.8 |
| Consumer/non-HCP | -1.9 | **+2.8*** | +0.6 | +0.3 |
| Unknown | -0.8 | +0.5 | -0.3 | -0.6 |

**Association: Reporter Type × Onset Time (χ² p < 0.001)**

| Reporter Type | 0–1 day | 2–7 days | 8–30 days | >30 days | Unknown |
| --- | --- | --- | --- | --- | --- |
| Physician | -1.2 | +0.8 | +1.4 | +0.6 | +0.9 |
| Pharmacist | **+2.4*** | -0.6 | -0.8 | -0.4 | -1.5 |
| Other HCP | +1.6 | +0.4 | +0.5 | +0.2 | -0.8 |
| Consumer/non-HCP | -0.5 | +1.2 | +0.8 | +0.5 | +0.4 |
| Unknown | -1.8 | -0.9 | -0.6 | -0.3 | **+2.6*** |

**Association: Reporter Type × Number of ADRs per Report (χ² p < 0.001)**

| Reporter Type | 1 ADR | 2 ADRs | 3 ADRs | 4 ADRs | ≥5 ADRs |
| --- | --- | --- | --- | --- | --- |
| Physician | -1.2 | -0.8 | **+2.5*** | +0.9 | +0.6 |
| Pharmacist | **+3.1*** | -1.5 | -1.2 | -0.8 | -0.5 |
| Other HCP | -1.8 | **+2.4*** | +0.6 | +0.9 | +0.4 |
| Consumer/non-HCP | -0.9 | +0.5 | **+2.2*** | +0.4 | +0.8 |
| Unknown | +0.6 | -0.4 | -0.8 | -0.6 | -0.3 |

**Supplementary Table S3: Adjusted Standardized Residuals (ASR) for Association Between Gender and ADR Variables**

**Association: Gender × Onset Time (χ² p = 0.007)**

| Gender | 0–1 day | 2–7 days | 8–30 days | >30 days | Unknown |
| --- | --- | --- | --- | --- | --- |
| Male | -1.4 | +0.5 | +0.3 | +0.2 | **+2.3*** |
| Female | **+2.2*** | -0.6 | -0.4 | -0.3 | -1.8 |
| Unknown | -0.8 | +0.3 | +0.2 | +0.1 | +0.5 |

**Association: Gender × Number of ADRs per Report (χ² p = 0.058, not significant)**

| Gender | 1 ADR | 2 ADRs | 3 ADRs | 4 ADRs | ≥5 ADRs |
| --- | --- | --- | --- | --- | --- |
| Male | -1.2 | +0.6 | +0.8 | **+2.1*** | +0.4 |
| Female | **+2.0*** | -0.5 | -0.7 | -1.6 | -0.3 |
| Unknown | -0.6 | +0.2 | +0.3 | +0.5 | +0.1 |

**Note:** Adjusted standardized residuals (ASR) with absolute values > 1.96 are considered statistically significant at p < 0.05 and are shown in **bold** with asterisks (***). Positive residuals indicate over-representation (observed count exceeds expected count under independence), while negative residuals indicate under-representation (observed count is less than expected). HCP = Healthcare Professional; ADR = Adverse Drug Reaction.
